# Accuracy of self-assessment of real-life functioning in schizophrenia

**DOI:** 10.1038/s41537-021-00140-9

**Published:** 2021-02-15

**Authors:** Paola Rocca, Claudio Brasso, Cristiana Montemagni, Silvio Bellino, Alessandro Rossi, Alessandro Bertolino, Dino Gibertoni, Eugenio Aguglia, Mario Amore, Ileana Andriola, Antonello Bellomo, Paola Bucci, Antonino Buzzanca, Bernardo Carpiniello, Alessandro Cuomo, Liliana Dell’Osso, Angela Favaro, Giulia Maria Giordano, Carlo Marchesi, Palmiero Monteleone, Lucio Oldani, Maurizio Pompili, Rita Roncone, Rodolfo Rossi, Alberto Siracusano, Antonio Vita, Patrizia Zeppegno, Silvana Galderisi, Mario Maj, the Italian Network for Research on Psychoses, Paola Bozzatello, Cristina Badino, Benedetta Giordano, Piergiuseppe Di Palo, Vitalba Calia, Marco Papalino, Stefano Barlati, Giacomo Deste, Anna Ceraso, Federica Pinna, Benedetta Olivieri, Daniela Manca, Giuseppe Piegari, Francesco Brando, Luigi Giuliani, Carmen Aiello, Laura Fusar Poli, Carmen Concerto, Teresa Surace, Mario Altamura, Stefania Malerba, Flavia Padalino, Pietro Calcagno, Martino Belvederi Murri, Andrea Amerio, Francesca Pacitti, Valentina Socci, Alessia Lucaselli, Laura Giusti, Anna Salza, Donatella Ussorio, Felice Iasevoli, Carla Gramaglia, Eleonora Gambaro, Eleonora Gattoni, Elena Tenconi, Enrico Collantoni, Paolo Meneguzzo, Paolo Ossola, Matteo Tonna, Maria Lidia Gerra, Claudia Carmassi, Barbara Carpita, Ivan Mirko Cremone, Giulio Corrivetti, Giammarco Cascino, Francesca Marciello, Roberto Brugnoli, Anna Comparelli, Valentina Corigliano, Nicoletta Girardi, Tommaso Accinni, Luca Carlone, Andrea Fagiolini, Arianna Goracci, Simone Bolognesi, Giorgio Di Lorenzo, Cinzia Niolu, Michele Ribolsi

**Affiliations:** 1 Department of Neuroscience, Section of Psychiatry, University of Turin, Turin, Italy; 2 Section of Psychiatry, Department of Biotechnological and Applied Clinical Sciences, University of L’Aquila, L’Aquila, Italy; 3 Department of Neurological and Psychiatric Sciences, University of Bari, Bari, Italy; 4 Department of Biomedical and Neuromotor Sciences, University of Bologna, Bologna, Italy; 5 Department of Clinical and Molecular Biomedicine, Psychiatry Unit, University of Catania, Catania, Italy; 6 Section of Psychiatry, Department of Neurosciences, Rehabilitation, Ophthalmology, Genetics and Maternal and Child Health, University of Genoa, Genoa, Italy; 7 Psychiatry Unit, Department of Medical Sciences, University of Foggia, Foggia, Italy; 8 Department of Psychiatry, University of Campania “Luigi Vanvitelli”, Naples, Italy; 9 Department of Neurology and Psychiatry, Sapienza University of Rome, Rome, Italy; 10 Section of Psychiatry, Department of Public Health, Clinical and Molecular Medicine, University of Cagliari, Cagliari, Italy; 11 Department of Molecular Medicine and Clinical Department of Mental Health, University of Siena, Siena, Italy; 12 Section of Psychiatry, Department of Clinical and Experimental Medicine, University of Pisa, Pisa, Italy; 13 Psychiatric Clinic, Department of Neurosciences, University of Padua, Padua, Italy; 14 Department of Neuroscience, Psychiatry Unit, University of Parma, Parma, Italy; 15 Department of Medicine, Surgery and Dentistry “Scuola Medica Salernitana” Section of Neuroscience, University of Salerno, Salerno, Italy; 16 Department of Psychiatry, University of Milan, Milan, Italy; 17 Department of Neurosciences, Mental Health and Sensory Organs, S. Andrea Hospital, Sapienza University of Rome, Rome, Italy; 18 Unit of Psychiatry, Department of Life, Health and Environmental Sciences, University of L’Aquila, L’Aquila, Italy; 19 Department of Systems Medicine, Psychiatry and Clinical Psychology Unit, Tor Vergata University of Rome, Rome, Italy; 20 Psychiatric Unit, School of Medicine, University of Brescia, Brescia, Italy; 21 Department of Mental Health, Spedali Civili Hospital, Brescia, Italy; 22 Department of Translational Medicine, Psychiatric Unit, University of Eastern Piedmont, Novara, Italy; 23 Department of Neuroscience, Reproductive Sciences and Dentistry, University of Naples Federico II, Naples, Italy; 24 Department of Mental Health of Salerno, Salerno, Italy

**Keywords:** Psychosis, Schizophrenia

## Abstract

A consensus has not yet been reached regarding the accuracy of people with schizophrenia in self-reporting their real-life functioning. In a large (*n* = 618) cohort of stable, community-dwelling schizophrenia patients we sought to: (1) examine the concordance of patients’ reports of their real-life functioning with the reports of their key caregiver; (2) identify which patient characteristics are associated to the differences between patients and informants. Patient-caregiver concordance of the ratings in three Specific Level of Functioning Scale (SLOF) domains (interpersonal relationships, everyday life skills, work skills) was evaluated with matched-pair *t* tests, the Lin’s concordance correlation, Somers’ *D*, and Bland–Altman plots with limits of agreement (LOA). Predictors of the patient-caregiver differences in SLOF ratings were assessed with a linear regression with multivariable fractional polynomials. Patients’ self-evaluation of functioning was higher than caregivers’ in all the evaluated domains of the SLOF and 17.6% of the patients exceeded the LOA, thus providing a self-evaluation discordant from their key caregivers. The strongest predictors of patient-caregiver discrepancies were caregivers’ ratings in each SLOF domain. In clinically stable outpatients with a moderate degree of functional impairment, self-evaluation with the SLOF scale can become a useful, informative and reliable clinical tool to design a tailored rehabilitation program.

## Introduction

Patients with schizophrenia show notable impairments in everyday functioning, including deficits in social, vocational, and residential domains, even during periods of remission from active psychosis^1^. Many different instruments are available for the assessment of real-life functioning, including rating scales that employ informant and self-reports^2^, direct observations by trained clinicians^3^, and performance-based measures^4^.

Studies have indicated that informant reports regarding the specific behaviors reflective of community functioning may be the most reliable assessment of functioning^5^. However, many people with schizophrenia do not have informants readily available to report on their functioning^6^ or they may have limited contact with them^7^. Also, in outpatient samples, there are many behaviors to which the clinician has no access and the use of self-reports may be important to get a clearer picture of the subjective level of functioning of patients. Nonetheless, self-reports of everyday functioning on the part of people with schizophrenia have been found to be poorly correlated with the reports of other informants and with their own performance of tests of cognition and functional abilities^8^.

Different studies have investigated the accuracy of self-appraisal in both clinical populations and healthy individuals. Healthy individuals tend to overestimate their abilities. In particular, poor performers showed a particularly positive bias, i.e., a tendency to overestimate their performance^9,10^. On the contrary, people with mild depressive symptoms tend to be more accurate in their self-evaluation^11^, with more severe depression symptoms associated with underestimation of functioning^7^. In studies of people with neurological conditions including multiple sclerosis^12^, traumatic brain injury^13^, mild cognitive impairment, and very mild Alzheimer disease^14^ similar results have been found: patients with poorer neuropsychological test performance tend to underestimate their impairment.

Similarly, people with schizophrenia have substantial problems in self-reporting everyday functioning^7^, as only one-third of chronic schizophrenia patients may be able to accurately report their functional abilities^5^. This is not surprising, as lack of insight is a prevalent feature of schizophrenia and is found across cultures^15,16^, in early and late^17,18^, acute and non-acute^19^ phases of the disorder. Poor insight in schizophrenia includes unawareness of symptoms, treatment need, psychosocial consequences of illness^20^, and alterations in cognitive processes, which involve the capacity for self-reflectiveness and resistance to excessive certainty^21,22^. This deficit is not just the consequence of a failure to notice a problem or accept a label but a failure to make consensually valid sense of complex and potentially traumatic experience, which limits patients’ abilities to form integrated sense of self^23^. This lack of insight likely has multiple roots, which include symptom severity, deficits in neurocognition, social cognition and metacognition, and sociopolitical factors^23^, and influences patients’ self-appraisal of their performance on objective tests^24,25^ and of their own levels of real-life functioning^6,7,26^. In particular, people with schizophrenia tend, on average, to underestimate the severity of their symptoms and to overestimate their psychosocial functioning^5^.

Moreover, analyses of variables influencing misestimation of self-reported real-life functioning showed that a higher level of positive symptoms and poorer cognitive and functional capacity associated with a tendency to overestimate real-life functioning^5^. Conversely, depression showed a unique relationship with real-life functioning, as it showed both adverse impacts on functioning and positive correlations with self-assessment abilities. Indeed, more severe depressive symptoms were associated with less overestimation in self-reports^5^ and with a higher degree of underestimation^7^. Besides depression, this pattern of personal appraisal is also potentially linked to insight and stigma. About this complex interplay, several studies found that self-stigma mediated the relationship between insight and depression^27–29^. Others showed that, beyond stigma, a generally negative appraisal of one’s future influences the effects of insight on mood^30^.

The purpose of this study was to examine the concordance of schizophrenia patients’ reports of their everyday life functional status with the reports of their informant and to identify which patient characteristics were associated with disagreement in these ratings between patients and caregivers.

We hypothesized that, based on previous studies, self-reported real-word functioning by people with schizophrenia would be poorly convergent with informants’ reports and that higher levels of cognitive performance and real-life functioning, rated by caregivers, would predict more concordant functioning ratings between patients and informants.

## Results

### Study population characteristics

The study population included 618 patients followed up in the 24 centers that participated in the second wave of the Italian Network for Research on Psychoses (NIRP) study between March 2016 and January 2018. Patients were all diagnosed with schizophrenia according to DSM-IV, mostly males (69.1%), aged on average 45.1±10.5 years and received 11.7±3.4 years of education. Only a minority of them lived in residential facilities (10.1%), and 34.4% were working. Antipsychotic treatment, mainly second-generation antipsychotics (69.3%), was administered to the vast majority of patients (97.4%); 54.4% were subject to polypharmacy, 34.3% to psychosocial intervention, 14.9% to psychotherapy, and 43.7% reported a relapse during the past 4 years. Psychiatric follow-up visits were scheduled monthly on about half of the patients (48.5%), whereas 17.4% of them needed a tighter control (Table 1). Illness-related factors, functional capacity, and real-life functioning mean scores are reported in detail in a previous paper^31^.

**Table 1:** Socio-demographic and clinical variables of the study population.

Gender (% males)	69.1
Age (years, mean ± SD)	45.1 ± 10.5
Education (years, mean ± SD)	11.7 ± 3.4
Working (%)	34.4
Currently in a residential facility (%)	10.1
Stable affective relationships (%)	18.9
*Current drug treatment*	
Antipsychotics (%)	97.4
First-generation (%)	13.1
Second-generation (%)	69.3
Both first- and second-generation (%)	15.0
Antidepressants (%)	17.6
Mood stabilizers (%)	26.0
Anxiolytics (%)	32.7
Anticholinergics (%)	9.4
Polypharmacy (%)	54.4
Any psychosocial interventions (%)	34.3
Psychotherapy (%)	14.9
Home care (%)	8.3
Relapse during past 4 years (%)	43.7
*Frequency of follow-up visits (%)*	
Monthly	48.5
Less than monthly	34.1
More than monthly	17.4

*SD* standard deviation

### Comparison between patient and caregiver evaluation of real-life functioning

Patients’ self-evaluation of functioning was higher than caregivers’ in all the evaluated items of the SLOF scale. Their mean scores were significantly different (Table 2) for all the items of the interpersonal relationship and work skills domains, and for most items of the everyday life skills. However, even if statistical significance was achieved, the mean difference between the paired scores was usually quite small in magnitude. Percent agreement and Gwet’s agreement’s coefficient were always high or very high: the smallest Gwet’s AC was 0.782 for the “Participates in groups” item of the interpersonal relationships domain, and it ranged 0.782–0.827 in interpersonal relationships, 0.813–0.958 in everyday life skills, and 0.793–0.866 in work skills. All probabilistic benchmark intervals were 0.600–0.800 or 0.800–1.000, thus indicating an at least substantial agreement between patient and caregiver. Also, when evaluating concordance on the three domains’ scores evaluated, we found that patients overestimated their functioning with respect to caregivers in all domains (Table 3 and 4). All paired differences were significant; however, the largest ones were barely over 1 point. Lin’s concordance ranged 0.766–0.874 and Somers’ *D* was between 0.25 and 0.27 (indicating a 25–27% probability that functioning scores self-assessed by patients exceeded those assessed by caregivers).

**Table 2:** Mean scores and concordance between patient and caregiver assessments of SLOF items.

Items interpersonal relationships	Patient mean score	Caregiver mean score	Patient-caregiver mean score difference	Wilcoxon matched-pair test on patient-caregiver mean score difference (*z*; *p* value)	Percent agreement	Gwet’s AC	Gwet’s AC probabilistic benchmark interval
13. Accept contact with others	3.55	3.39	0.15	5.07; <0.001	0.941	0.827	0.800–1.000
14. Initiate contact with others	3.17	3.07	0.11	3.75; <0.001	0.940	0.814	0.600–0.800
15. Communicate effectively	3.80	3.62	0.18	5.70; <0.001	0.943	0.833	0.800–1.000
16. Engages in activities without prompting	3.21	3.05	0.16	4.85; <0.001	0.938	0.799	0.600–0.800
17. Participates in groups	2.91	2.78	0.13	3.83; <0.001	0.936	0.782	0.600–0.800
18. Forms and maintains friendships	2.87	2.72	0.16	4.63; <0.001	0.938	0.802	0.600–0.800
19. Asks for help when needed	3.70	3.56	0.14	3.62; <0.001	0.935	0.805	0.600–0.800

Items everyday life skills	Patient mean score	Caregiver mean score	Patient-caregiver mean score difference	Wilcoxon matched-pair test on patient-caregiver mean score difference (*z*; *p* value)	Percent agreement	Gwet’s AC	Gwet’s AC probabilistic benchmark interval
27. Household responsibilities	3.68	3.53	0.15	5.63; <0.001	0.948	0.837	0.800–1.000
28. Shopping	3.93	3.84	0.09	3.84; <0.001	0.951	0.862	0.800–1.000
29. Handling personal finances	3.60	3.45	0.15	5.55; <0.001	0.943	0.813	0.600–0.800
30. Use of telephone	4.55	4.51	0.04	2.35; 0.019	0.971	0.952	0.800–1.000
31. Traveling from residence without getting lost	4.55	4.48	0.07	2.76; 0.006	0.975	0.958	0.800–1.000
32. Use of public transportation	4.30	4.25	0.05	2.89; 0.004	0.972	0.944	0.800–1.000
33. Use of leisure time	4.17	4.02	0.15	5.27; <0.001	0.952	0.878	0.800–1.000
34. Recognizing and avoiding common dangers	4.52	4.46	0.06	2.97; 0.003	0.972	0.949	0.800–1.000
35. Self-medication	4.17	4.00	0.17	5.64; <0.001	0.955	0.889	0.800–1.000
36. Use of medical and other community services	4.28	4.15	0.13	5.33; <0.001	0.964	0.916	0.800–1.000
37. Basic reading; writing and arithmetic	4.52	4.50	0.02	0.98; 0.330	0.967	0.940	0.800–1.000

Items work skills	Patient mean score	Caregiver mean score	Patient-caregiver mean score difference	Wilcoxon matched-pair test on patient-caregiver mean score difference (*z*; *p* value)	Percent agreement	Gwet’s AC	Gwet’s AC probabilistic benchmark interval
38. Has employable skills	3.11	2.97	0.14	4.65; <0.001	0.946	0.814	0.600–0.800
39. Works with minimal supervision	3.10	2.95	0.15	5.06; <0.001	0.941	0.793	0.600–0.800
40. Is able to sustain work effort	2.92	2.79	0.13	4.98; <0.001	0.945	0.812	0.600–0.800
41. Appears at appointments on time	4.04	3.92	0.13	4.42; <0.001	0.950	0.866	0.800–1.000
42. Follows verbal instructions accurately	3.90	3.78	0.12	4.56; <0.001	0.947	0.846	0.800–1.000
43. Completes assigned tasks	3.82	3.66	0.17	5.87; <0.001	0.945	0.834	0.800–1.000

**Table 3:** Mean scores and concordance between patient and caregiver assessments of SLOF domains.

	Patient mean score	Caregiver mean score	Patient-caregiver mean score difference (95% CI)	Paired *t* test on patient-caregiver mean score difference (*t*; *p* value)	Lin’s concordance correlation	Somers’ *D* (95% CI)
Interpersonal relationships	23.23	22.19	1.04 (0.72–1.36)	6.36; <0.001	0.766	0.250 (0.185–0.312)
Everyday life skills	46.22	45.19	1.03 (0.66–1.39)	5.50; <0.001	0.874	0.270 (0.209–0.328)
Work skills	20.90	20.07	0.83 (0.56–1.11)	6.00; <0.001	0.834	0.265 (0.204–0.323)

**Table 4:** Multivariable regressions of patient-caregiver discrepancies in SLOF domains.

	Patient-caregiver discrepancies in SLOF domains		
	Interpersonal relationships (*n* = 613)	Everyday life skills (*n* = 608)	Work skills (*n* = 612)
Caregiver score	−0.295 (−0.351; −0.239) <0.001	−0.244 (−0.288; −0.200) <0.001	−0.182 (−0.219; −0.144) <0.001*
PANSS positive	−0.116 (−0.190; −0.042) 0.002		
PANSS disorganization		−0.798 (−1.171; −0.425) <0.001*	
BNSS avolition	−0.264 (−0.373; −0.156) <0.001		−0.185 (−0.276; −0.094) <0.001
BNSS expressive deficits		−0.529 (−0.809; −0.249) <0.001*	
Processing speed			0.019 (0.006; 0.031) 0.005
Constant	1.042 (0.746; 1.339) <0.001	1.602 (1.223; 1.981) <0.001	1.233 (0.965; 1.502) <0.001

Note: multivariable regressions performed using multiple imputations and multivariable fractional polynomials (MFP). Linear regression coefficients are displayed with 95% confidence intervals in brackets and *p* values. * denotes cubic regression coefficients.

**Fig. 1:**
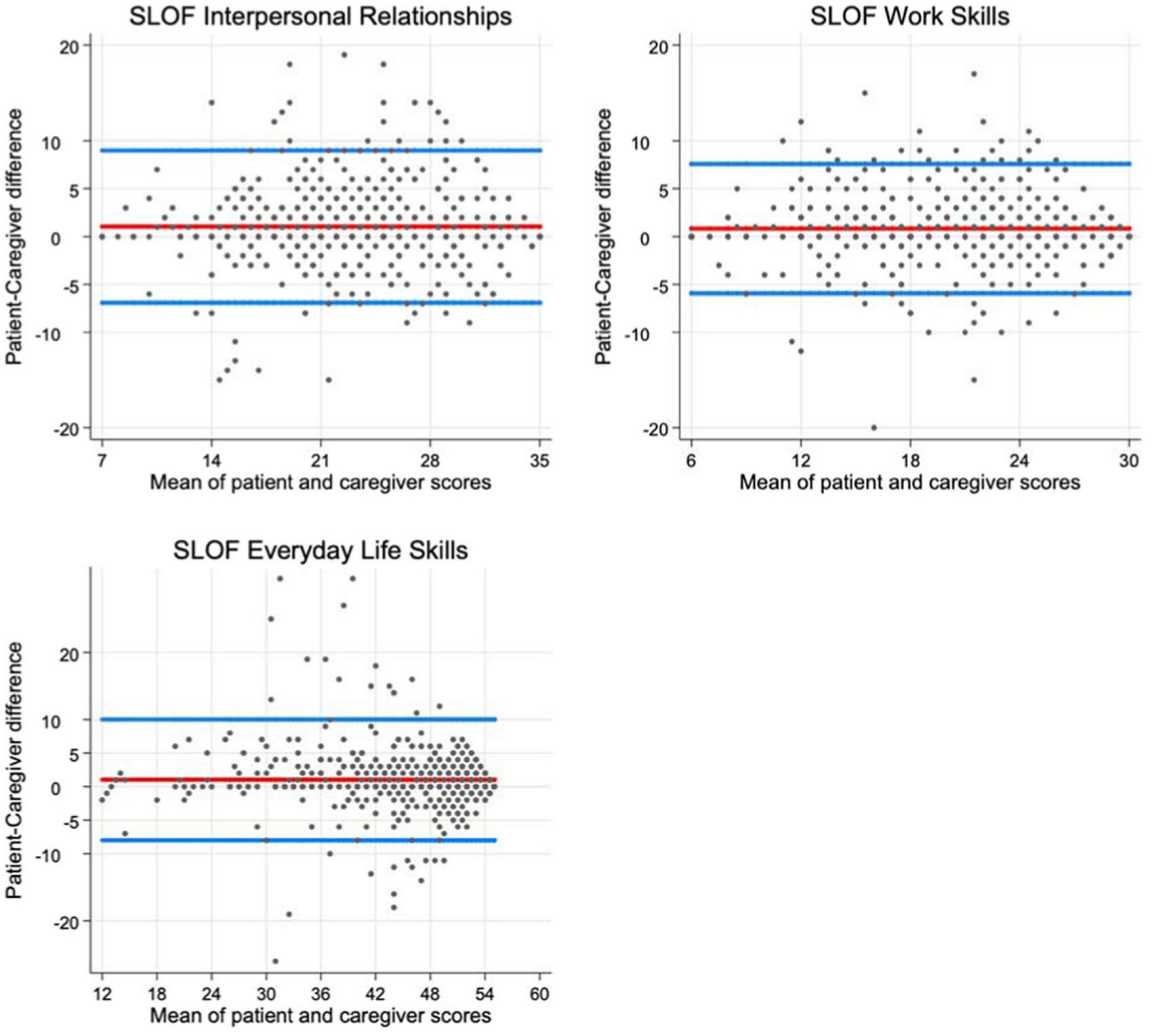
Bland–Altman plots of patient-caregiver agreement on SLOF domains. Note: in these Bland–Altman plots circles represent patients; the upper and lower blue lines represent the 95% limits of agreement; the red line represents the average discrepancy between patients and caregivers. Cases lying between the blue lines are those whose patient and caregiver scores were with 95% probability concordant; those lying over the upper blue line are those whose self-reported functioning score was significantly higher than the corresponding score attributed by the caregiver; cases lying below the upper blue line are those whose self-reported functioning score was significantly lower than the corresponding score attributed by the caregiver. In the *x* axes, the mean of patient and caregiver scores is assumed as the more likely value of patient’s functioning.

The Bland–Altman plots (Fig. 1) confirmed the high concordance of patient and caregiver assessments, as most subjects were placed within the limits of agreement (LOA), for all three domains. Those who lied outside the LOA were distributed quite randomly, only slightly more prevalent in the upper part of the graph (patients’ scores higher than caregivers’ scores) and in the central part of the mean total range (average values of functioning). These patients overall account for 17.6% of the study population (*n* = 83); 13.4% exceeded LOA in only one SLOF domain, 2.1% exceeded LOAs in two SLOF domains, and 2.1% in all the three SLOF domains.

### Predictors of patient-caregiver discrepancies in the evaluation of real-life functioning

**Fig. 2:**
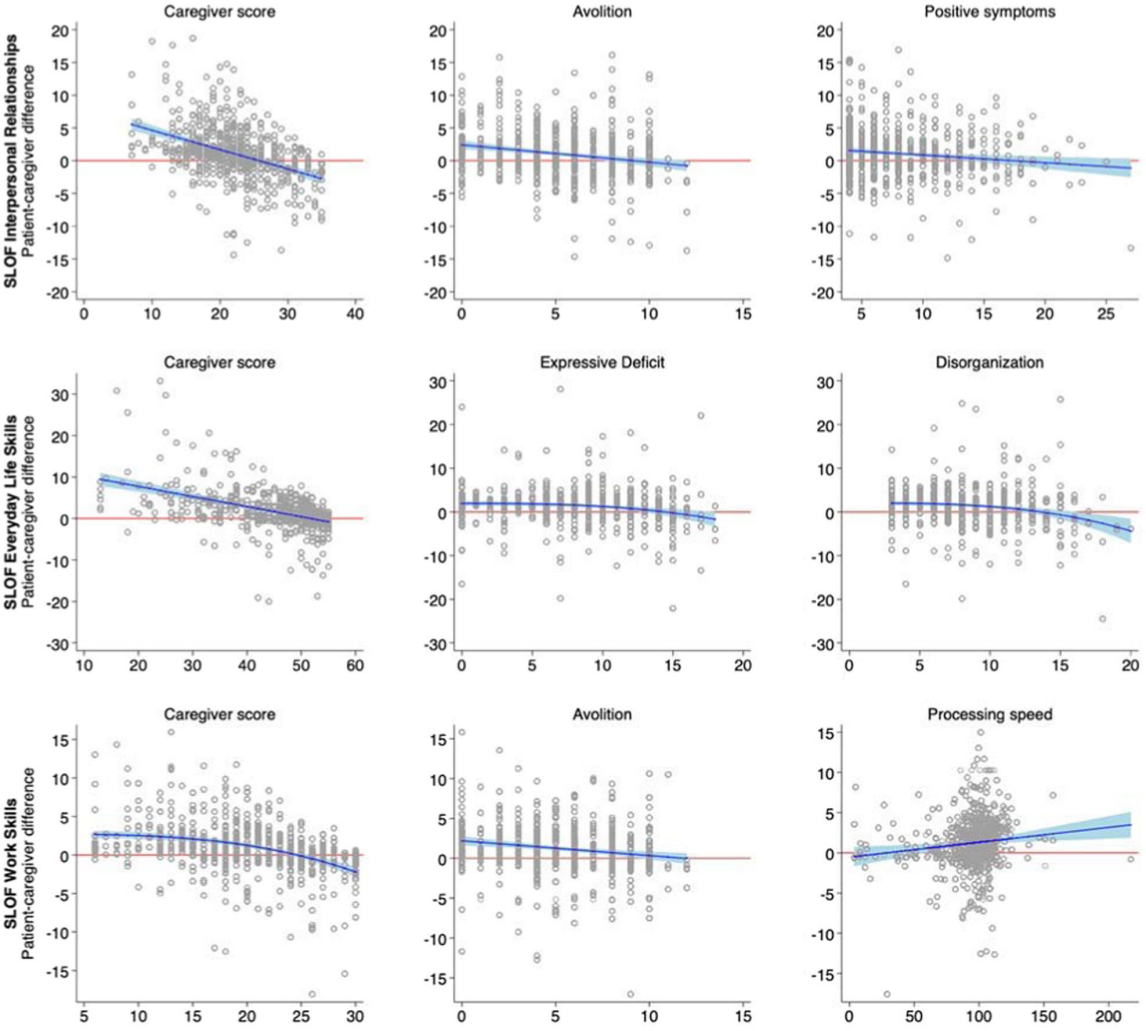
Scatterplots of patient-caregiver differences in the three SLOF domains and their significant predictors, with the estimated regression curve. Note: in these plots, circles represent patients, whose coordinates are given by patient-caregiver difference (*Y* axis) and values of its significant predictors according to multivariable fractional polynomial regression (*X* axis). The blue line represents the estimated regression curve, with 95% confidence interval as shaded area.

From the regression analyses performed using multiple imputation and multivariable fractional polynomials (MFPs, Table 4 and Fig. 2), we found that caregiver scores in each of the three domains of the SLOF analyzed were the strongest predictors of patient-caregiver discrepancies with negative coefficients. This indicates that patients’ overestimation was related to low caregiver’s scores; patient’s estimation was less discrepant when caregiver scores were higher and for the Interpersonal Relationships and Work Skills domains at higher caregiver scores corresponded underestimation of patients. Avolition was associated with the patient-caregiver discrepancy in the evaluation of interpersonal relationships and, to a lesser extent, of work skills: more severe avolition was associated with more precise patient’s self-evaluations. Expressive deficits were associated with patient-caregiver discrepancy in work skills, positive symptoms with patient-caregiver discrepancy in interpersonal relationships, and disorganization symptoms with patient-caregiver discrepancy in everyday life skills domain with a similar negative relation, i.e., more severe symptoms were associated with patient’s self-evaluations closer to caregivers’ ones. Finally, higher speed of processing in the MCCB was poorly associated with a higher level of patient’s overestimation in the work skills domain.

## Discussion

This study aimed at two main goals: (a) to assess the concordance between real-world functioning self-reported by people with schizophrenia and reports generated by informants; (b) to evaluate predictors of the agreement between these two evaluations.

To our knowledge, this is the largest study carried out so far examining awareness of functional deficits in people with schizophrenia. Subjects participating in this study were living in the community and stabilized on antipsychotic treatment and were evaluated for their everyday life skills, interpersonal abilities, and work skills.

Concerning the first aim, our results demonstrated good concordance between self-reported functioning and informant reports, contrary to our research hypothesis. About 5/6 of patients self-evaluated their real-life functioning in concordance with their caregivers’ estimation, whereas only 17.6% of patients were poorly concordant, mostly on one single domain of functioning. These data suggest that patients had few problems in reporting their real-life functioning and that self-report could be informative and should be considered to design rehabilitation projects.

This finding is in contrast with previous studies showing that more than half of patients were inaccurate raters of their abilities^5,7,8,32^. These contrasting results may be owing to differences in the samples and in the types of informants. First, our patients were community-dwelling clinically stable patients, with a good service engagement and a moderate degree of functional impairment^31^. Moreover, about 2/3 of them underwent scheduled follow-up visits at least once every month for 4 years, which suggests a stable course of the illness and confirms a high service engagement that might have contributed to our patients’ good awareness about their own real-life functioning and to the high concordance with informants’ ratings. In other terms, as shown by a recent study presented by Vohs et al.^33^, we can assume that this meaningful engagement with mental health services could improve patients’ ability to appraise the consequences of the disorder they suffer from. Moreover, this enhanced level of cognitive and clinical insight could have led a considerable portion of the patients in our sample to recovery, intended as a meaningful interpretation of the challenges the disorder poses and a possibility to meaningfully live with schizophrenia^34^. As regards the informant, in our study and in the Italian context it was mostly a key caregiver, the individual most frequently and closely in contact with the patient, which shares many time and activities with patients. This may have led to a greater concordance of their viewpoints about patients’ real-life functioning, not in line with previous studies that chose the high contact clinical as informant^5,7,8,32^. We hypothesize that this type of social context influences both the patient, who is constantly made aware of her/his functional limits by the caregiver and the caregiver, who is able to accurately evaluate the patient’s functioning in the wake of her/his continuous relationship with the patient. Moreover, as they share similar roles in patients’ lives and therefore similar point of view, the choice of key caregivers as informants entails a high level of homogeneity among different caregivers’ evaluations. Accordingly, unlike other studies where informants’ evaluations where performed by non-clinicians who have different roles in the patients’ lives (e.g., key caregivers, close relatives, and friends simultaneously in the same sample), we did not expect a high degree of variability depending on the informants’ role in the patients’ lives.

About the second aim, different types of errors may occur when asking people with schizophrenia to self-rate their functional skills, including misunderstanding the items on a rating scale, inaccurately conceptualizing normal functioning, and making inaccurate comparisons to external standards. These types of errors could derive from cognitive deficits, from the severity of specific symptomatic dimensions, and from the socio-demographic characteristics of the patients. Identifying which variables were associated with self-rated real-life functioning misestimation can be useful to determine which patients are more likely to give a biased self-evaluation.

The strongest predictors of patient-caregiver discrepancies in all the three SLOF domains were caregivers’ scores. This is an expected result, because the magnitude of patient-caregiver disagreement is at least partly constrained by the reference value represented by the caregiver score; as such, in our regression models, the caregiver score was essential to adjust the predictors’ coefficient estimates. Patients who received higher ratings from caregivers showed better concordance with the caregiver’s evaluation. This finding is consistent with our research hypothesis and with previous studies showing that patients who have poorer functioning assessed by the informant tend to overestimate their own functioning^5,7,8^. However, in our study inaccuracy, measured by Bland–Altman LOA, affected only 1/6 of the patients, which is a much smaller proportion compared with previous studies.

As to symptoms, at odds with previous studies^5,7^, we did not find any association between self-rating of real-world functioning and depressive symptoms; instead we found that the avolition dimension of negative symptoms was the main predictor after the caregivers’ scores, with more severe avolition associated with a better concordance or even with an underestimation of the SLOF Interpersonal domain functioning in patient’s self-ratings. We hypothesize that these results may depend on the different instruments utilized to assess depression and negative symptoms: while we utilized an objective (based on clinicians’ observations during the interview) schizophrenia specific depression rating scale, the Calgary Depression Scale for Schizophrenia^35^ (CDSS), previous studies employed a self-reported scale, namely the Beck Depression Inventory version I or II^5,7,32,36,37^. Moreover, the BDI-II includes items concerning anhedonia, asociality and avolition, which overlap with the experiential domain of negative symptoms that we evaluated with the Brief Negative Symptoms Scale (BNSS)^38^. Similarly, according to the current conceptualization of negative symptoms^39^, we assessed negative symptoms with a specific second-generation assessment scales, the BNSS, considering two factors: avolition and expressive deficits^40,41^, whereas previous studies did not employ specific scales and considered negative symptoms as a single variable^7,32,37,42–44^. These differences in clinical assessment could at least in part account for our contrasting results compared with previous works. We suppose that the avolition dimension of the BNSS, as experiential domain of negative symptoms including avolition, anhedonia, and asociality, similarly to depression in other studies^45,46^, can support a self-evaluation of real-life functioning similar to the one provided by the caregiver. In other words, a higher degree of avolition could enhance a more “objective” perception of patients’ limits of functioning.

The other significant predictors of discrepancies in the SLOF dimensions evaluation were illness-or cognitive-related factors that generally had weak associations with the outcome (cf. Table 3). As we found high concordance in all domains, a weak impact of predictors on disagreement might be expected, because there is little variance of disagreement to be explained, especially after adjusting for the caregivers’ score. Moreover, the large size of the study population might have enhanced statistical significance even for relatively small effects, which may also be influenced by the presence of few subjects with extreme values of discrepancy. For all these reasons, the substantive significance of these associations seems quite poor and should not be emphasized. Thus, the absent or weak association of cognitive performance with disagreement on functioning assessment was an unexpected result that disconfirmed our research hypothesis.

The patients included in the present study were outpatients with stable symptoms, moderate degree of functional impairment, and a strong and stable relationship with mental services and their caregiver. These characteristics could have helped in achieving a high level of agreement among patients’ and caregivers’ evaluation. Therefore, the main limitation of the present study is that our results may not be reproducible in patients in acute phases, clinically unstable, or assessed in other clinical settings or in social context were a key caregiver is absent. In addition, the high degree of agreement made it difficult to identify predictors of patient-caregiver discrepancy, beside the functioning level itself.

Despite this limitation, this study has some important strengths: the large sample size, the naturalistic design without selection bias related to randomized controlled designs, the use of state-of-the-art statistical analysis and instruments to assess psychopathology, cognition, functional capacity, and real-world functioning.

In our sample, people with schizophrenia showed a good agreement with the caregivers’ ratings of the SLOF scale. It can be assumed that this good level of concordance is partly owing to sample characteristics: clinically stable, non-hospitalized patients, with moderate degree of functional impairment, who often share background and life context with their caregivers. Moreover, a good service engagement with continuative community-based psychiatric care might enhance patients’ metacognitive reflection, insight, and ability to self-appraise their real-life functioning. In this view, in contexts in which community-based mental health care is provided, and in outpatients with clinically stable schizophrenia and a good engagement with mental health services, self-evaluation with the SLOF scale can become a useful, informative and reliable clinical tool to design a tailored rehabilitation program.

## Methods

### Participants

We used the 4-year follow-up database of the NIRP study, involving at the baseline 921 community-dwelling, clinically stable patients^47,48^. Twenty-four out of the 26 Italian university psychiatric clinics and/or mental health departments that joined the baseline study participated in the follow-up. All patients recruited by the 24 participating centers for the baseline study were invited to participate.

Patients with a diagnosis of schizophrenia according to the DSM-IV and confirmed with the Structured Clinical Interview for DSM-IV-Patient version (SCID-I-P) were included in the follow-up study. Exclusion criteria were (a) a history of head trauma with loss of consciousness in the 4-year interval between the baseline and the follow-up assessments; (b) progressive cognitive deterioration possibly owing to dementia or other neurological illness diagnosed in the last 4 years; (c) a history of alcohol and/or substance abuse in the last six months; (d) current pregnancy or lactation; (e) inability to provide informed consent, and (f) treatment modifications and/or hospitalization owing to symptom exacerbation in the last three months.

For the participants in the baseline study who could not be traced or were deceased, investigators had to fill in a specific form reporting clinical information available at the last contact and, if available, the cause of death in case of decease. After receiving a comprehensive explanation of the study procedures and goals, a written informed consent to participate in the follow-up procedures was asked to all patients. The authors assert that all procedures contributing to this work comply with the ethical standards of the relevant national and institutional committees on human experimentation and with the Helsinki Declaration of 1975, as revised in 2008. All procedures involving human patients were approved by local Ethics Committees of the participating centers, and recruitment was carried out from March 2016 to December 2017.

### Procedures

The assessments of the enrolled patients were completed following the same schedule used in the baseline study^47^: (1) socio-demographic information, psychopathological, and neurological assessments on the first day; (2) neurocognitive, social cognition, and functional capacity assessments on the second day; (3) according to the patient’s preference, assessment of personal resources and perceived stigma were carried out on the third day morning, or in the afternoon of any of the days. For real-life functioning assessment, the patient’s key caregiver, was invited to join one of the scheduled sessions.

### Evaluation of illness-related factors

A clinical form was filled in with information on disease course and treatments in the previous 4 years, using all available sources of information (patients, relatives, medical records, and mental health workers).

The Positive and Negative Syndrome Scale (PANSS)^49^ was used to rate symptom severity. Positive symptoms were assessed using four items of the PANSS: P1(delusions), P3 (hallucinatory behavior), P5 (grandiosity), G9 (unusual thought content). Disorganization was assessed using three items of the PANSS scale: P2 (conceptual disorganization), N5 (difficulty in abstract thinking), and G11 (poor attention). We used the consensus 5-factor solution proposed by Wallwork et al.^50^; for both dimensions, higher scores represent greater symptom severity. Negative symptoms were assessed using the Italian version of the Brief Negative Symptom Scale (BNSS)^38^; the scores of the two domains Avolition (sum of anhedonia, asociality, and avolition) and expressive deficit (sum of blunted affect and alogia) were used in statistical analyses (higher scores correspond to greater severity)^40,41^. Depressive symptoms were evaluated by the CDSS^35^; the total score was used in data analyses (higher scores correspond to greater severity of depression).

The Measurement and Treatment Research to Improve Cognition in Schizophrenia (MATRICS) Consensus Cognitive Battery (MCCB)^51^ was used to assess the following neurocognitive domains: processing speed, attention/vigilance, working memory, verbal learning, visual learning, and reasoning and problem solving (for all domains, higher scores represent better cognitive functioning). The assessment of social cognition was partly included in the MCCB (Mayer–Salovey–Caruso Emotional Intelligence Test, MSCEIT, managing emotion section)^52^, which examines the regulation of emotions in oneself and in one’s relationships with others by presenting vignettes of various situations, along with ways to cope with the emotions depicted in these vignettes. This test was integrated by the Facial Emotion Identification Test^53^, measuring emotion recognition, and The Awareness of Social Inference Test (TASIT)^54^, which is a theory of mind (ToM) test consisting of seven scales (positive emotions, negative emotions, sincere, simple sarcasm, paradoxical sarcasm, sarcasm enriched, lie), organized into three sections: emotion recognition; social inference (minimal); social inference (enriched).

### Assessment of personal resources

The Resilience Scale for Adults^55^, a self-administered scale, was used to assess perception of self, perception of the future, social competence and family cohesion (higher scores correspond to higher resilience). The Service Engagement Scale^56^ was employed to measure patients’ levels of difficulty to engage with mental health services (higher total score represents greater difficulty).

### Evaluation of context-related factors

The Internalized Stigma of Mental Illness^57^ questionnaire evaluated the experience of internalized stigma (higher total score corresponds to greater experience of internalized stigma). The number of incentives was registered as a count variable, ranging from 0 to 4, and includes the availability of a disability pension, access to family practical and financial support, and registration in the unemployment list.

### Assessment of functional capacity and real-life functioning

The short version of the University of California San Diego (UCSD) Performance-based Skills Assessment Brief (UPSA-B)^58^ was used to assess functional capacity. It assesses “financial skills” and “communication skills”. Participants receive scaled scores for each of the subscales (range = 0–50), which are summed to create an overall score ranging from 0 to 100. Higher scores indicate better functional capacity.

Real-life functioning was evaluated using the Specific Level of Functioning Scale (SLOF, Italian version)^59–61^ a hybrid instrument that explores many aspects of functioning. It consists of a 43-item self- or informant-rated scale of a person’s behavior and functioning, which assesses the following domains: physical efficiency, skills in self-care, interpersonal relationships, social acceptability, everyday life skills (e.g., shopping, using public transportation), and working skills. Each of the 43 items are rated on a five-point Likert scale, indicating the level of assistance the participant needs to perform the task, with higher score indicating better functioning. The SLOF scale differs from the other outcome measures in emphasizing patient’s current functioning and observable behavior, as opposed to inferred mental or emotional states, and focuses on a person’s skills, assets, and abilities rather than deficits. Moreover, the SLOF does not include items relevant to psychiatric symptomatology or cognitive dysfunctions. SLOF interpersonal relationships, everyday life skills, and work skills domains were included in statistical analyses. In this follow-up study, the patients her/himself and her/his key caregiver separately answered to the 43 SLOF items. The patient’s key caregiver, preferably the same interviewed in the baseline study, was chosen as informer as usually, this is the individual most frequently and closely in contact with the patient in the Italian context.

### Training of researchers and inter-rater reliability

Researchers were trained by the coordinating center two months before the beginning of the follow-up recruitment to ensure consistency with the baseline data collection procedures. The inter-rater reliability was evaluated by Cohen’s kappa for categorical variables, and intraclass correlation coefficient (ICC) for continuous variables. For items showing a small degree of variation among patients, the percentage of perfect agreement was calculated. An excellent inter-rater agreement was found for the SCID-I-P (Cohen’s kappa = 0.91). Good to excellent agreement was observed for BNSS (ICC = 0.74–0.97), PANSS (ICC = 0.60–0.98, percentage agreement = 64–100%), CDSS (ICC = 0.76–0.98), and MCCB (ICC = 0.98). Further details can be found in Galderisi et al.^31^.

### Statistical analysis

The characteristics of the study population were summarized by reporting continuous variables as mean ± standard deviation and categorical variables as percentages. Patient-caregiver agreement was first calculated on each of the 24 items belonging to the interpersonal, activities, and work domains of the SLOF scale, and subsequently on these three domains. As the SLOF items are ordinal variables on the 1–5 range, agreement on the items’ level was assessed by reporting the patients’ and caregivers’ mean score and their difference, the two-sided Wilcoxon matched-pair test of the null hypothesis of equality of means, the percentage agreement, the Gwet’s agreement coefficient (AC) and its related probabilistic benchmark interval. Gwet’s AC coefficient is recently preferred to Cohen’s kappa family of coefficients because it has been demonstrated to be more robust, especially in the presence of skewed data, and to be able to avoid the paradox of negative agreement^62–65^. Moreover, Gwet’s ACs are categorized into the scheme provided by Landis and Koch^66^ as slight (0.00–0.20), fair (0.21–0.40), moderate (0.41–0.60), substantial (0.61–0.80), and almost perfect (0.81–1.00) using a probabilistic assignment, that takes into account the variance of the estimate^38^. We applied ordinal weighting to Gwet’s AC calculation in order to assign an increasing penalty when disagreement between raters was larger. The three domains of the SLOF are discrete-continuous variables, therefore for these variables the patient-caregiver agreement was assessed by reporting the patients’ and caregivers’ mean score and their difference, the two-sided matched-pair *t* test of the null hypothesis of equality of means, the Lin’s concordance correlation and Somers’s *D*, which assigns the probability that patients are less or more likely to overestimate their caregiver’s evaluation on the same domain. The Bland–Altman plots and LOA for each domain were also obtained, to understand whether disagreement between patient and caregiver occurred at particular values along the domains’ score range.

Finally, to investigate which characteristics could predict disagreement, we performed multivariable linear regressions of the patient-caregiver score difference for each SLOF domain on a set of covariates including gender, age, education, positive symptoms, disorganization, avolition, expressive deficit, depression, the six MCCB items of neurocognition, the five items of social cognition and functional capacity, adjusted for the caregiver’s score. These regression models were carried out using MFP on 10 imputed data sets obtained through multiple imputation of predictors’ missing data using chained equations. MFP procedure verified which functional form (linear or nonlinear, i.e., quadratic, cubic, square root, etc.) of each variable best represented its relationship with the outcome, and simultaneously selected those significantly associated to the outcome. Stata 15.1 was used for all analyses, specifically the user-written procedures kappaetc^63^, concord^67^, scsomersd^68^, and mfpmi^69^ allowed to estimate Gwet’s AC, Lin’s concordance correlation, Somers’ *D* and MFPs, respectively.

### Reporting summary

Further information on research design is available in the Nature Research Reporting Summary linked to this article.

## Supplementary information

REPORTING SUMMARY

## Data Availability

The data that support the findings of this study are available on request from the corresponding author. The data are not publicly available as they contain information that could compromise the privacy of research participants.

## Code availability

The code that supports the findings of this study is publicly available at: https://github.com/dingib/NIRP/blob/main/SLOF_concordance.do
