# Supplementary material for: Accuracy of self-assessment of real-life functioning in schizophrenia
Source: NPJ Schizophr. 2021 Feb 15;7:11. doi: 10.1038/s41537-021-00140-9 (PMC7884703; doi:10.1038/s41537-021-00140-9)
Supplement: Supplementary file 1 — REPORTING SUMMARY [file 41537_2021_140_MOESM1_ESM.pdf]

nature research

Corresponding author(s): Paola Rocca

Last updated by author(s): Nov 29, 2020

Reporting Summary

Nature Research wishes to improve the reproducibility of the work that we publish. This form provides structure for consistency and transparency in reporting. For further information on Nature Research policies, see our Editorial Policies and the Editorial Policy Checklist.

Statistics

For all statistical analyses, confirm that the following items are present in the figure legend, table legend, main text, or Methods section.

n/a	Confirmed	
☐	☒	The exact sample size (*n*) for each experimental group/condition, given as a discrete number and unit of measurement
☐	☒	A statement on whether measurements were taken from distinct samples or whether the same sample was measured repeatedly
☐	☒	The statistical test(s) used AND whether they are one- or two-sided<br>*Only common tests should be described solely by name; describe more complex techniques in the Methods section.*
☐	☒	A description of all covariates tested
☐	☒	A description of any assumptions or corrections, such as tests of normality and adjustment for multiple comparisons
☐	☒	A full description of the statistical parameters including central tendency (e.g. means) or other basic estimates (e.g. regression coefficient) AND variation (e.g. standard deviation) or associated estimates of uncertainty (e.g. confidence intervals)
☐	☒	For null hypothesis testing, the test statistic (e.g. *F*, *t*, *r*) with confidence intervals, effect sizes, degrees of freedom and *P* value noted<br>*Give P values as exact values whenever suitable.*
☒	☐	For Bayesian analysis, information on the choice of priors and Markov chain Monte Carlo settings
☒	☐	For hierarchical and complex designs, identification of the appropriate level for tests and full reporting of outcomes
☐	☒	Estimates of effect sizes (e.g. Cohen's *d*, Pearson's *r*), indicating how they were calculated

Our web collection on statistics for biologists contains articles on many of the points above.

Software and code

Policy information about availability of computer code

Data collection	No software code was used specifically to collect data for this study
Data analysis	Stata v.15.1 custom code available at: https://github.com/dingib/NIRP/blob/main/SLOF_concordance.do

For manuscripts utilizing custom algorithms or software that are central to the research but not yet described in published literature, software must be made available to editors and reviewers. We strongly encourage code deposition in a community repository (e.g. GitHub). See the Nature Research guidelines for submitting code & software for further information.

Data

Policy information about availability of data

All manuscripts must include a data availability statement. This statement should provide the following information, where applicable:

- Accession codes, unique identifiers, or web links for publicly available datasets
- A list of figures that have associated raw data
- A description of any restrictions on data availability

The data that support the findings of this study are available on request from the corresponding author. The data are not publicly available as they contain information that could compromise the privacy of research participants

Field-specific reporting

Please select the one below that is the best fit for your research. If you are not sure, read the appropriate sections before making your selection.

☐ Life sciences ☒ Behavioural & social sciences ☐ Ecological, evolutionary & environmental sciences

For a reference copy of the document with all sections, see nature.com/documents/nr-reporting-summary-flat.pdf

Behavioural & social sciences study design

All studies must disclose on these points even when the disclosure is negative.

Study description	Quantitative cross-sectional study
Research sample	618 patients followed up in the 24 centers that participated to the second wave of the Italian Network for Research on Psychoses (NIRP) study between March 2016 and January 2018. Patients were all diagnosed with schizophrenia according to DSM-IV, mostly males (69.1%), aged on average 45.1 years (SD = 10.5 y) and received 11.7 years (SD = 3.4 y) of education.
Sampling strategy	No statistical sampling procedure was applied. Patients who participated in the first wave of the study (which included all patients consecutively seen at the outpatient units of the participating centers) and that were available at 4-year follow-up constitute the research sample. The sample size was deemed sufficient because in multivariable analyses a moderate number of covariates compared to the number of subjects was used.
Data collection	Pen and paper were used to collect clinical data and to fill the scales employed. Data were then transferred into the online database. Patients and caregivers were with the reasearcher only when collecting data. Researchers were blind to the study hypothesis.
Timing	From March 2016 to December 2017.
Data exclusions	No data were excluded from the analysis.
Non-participation	No participants dropped out.
Randomization	Participants were not allocated into experimental groups.

Reporting for specific materials, systems and methods

We require information from authors about some types of materials, experimental systems and methods used in many studies. Here, indicate whether each material, system or method listed is relevant to your study. If you are not sure if a list item applies to your research, read the appropriate section before selecting a response.

Materials & experimental systems

n/a	Involved in the study	
☒	☐	Antibodies
☒	☐	Eukaryotic cell lines
☒	☐	Palaeontology and archaeology
☒	☐	Animals and other organisms
☐	☒	Human research participants
☐	☒	Clinical data
☒	☐	Dual use research of concern

Methods

n/a	Involved in the study	
☒	☐	ChIP-seq
☒	☐	Flow cytometry
☒	☐	MRI-based neuroimaging

Human research participants

Policy information about studies involving human research participants

Population characteristics	See above.
Recruitment	The patients included in the present study were outpatients with stable symptoms, moderate degree of functional impairment and a strong and stable relationship with mental services and their caregiver. Therefore our results may not be reproducible in patients in acute phases, clinically unstable, or assessed in other clinical settings or in social context were a key caregiver is absent.
Ethics oversight	Approval of the study protocol was obtained from the Local Ethics Committees of the participating centers.

Note that full information on the approval of the study protocol must also be provided in the manuscript.

Clinical data

Policy information about clinical studies

All manuscripts should comply with the ICMJE guidelines for publication of clinical research and a completed CONSORT checklist must be included with all submissions.

Clinical trial registration	Not a clinical trial
Study protocol	Not a clinical trial
Data collection	Not a clinical trial
Outcomes	Not a clinical trial
